# Complex Periodicity and Synchronization

**DOI:** 10.3389/fphys.2020.563068

**Published:** 2020-09-30

**Authors:** Korosh Mahmoodi, Bruce J. West, Paolo Grigolini

**Affiliations:** ^1^Department of Social and Decision Sciences, Carnegie Mellon University, Pittsburgh, PA, United States; ^2^Office of the Director, Army Research Office, Research Triangle Park, Durham, NC, United States; ^3^Center for Nonlinear Science, University of North Texas, Denton, TX, United States

**Keywords:** reinforcement learning, complex adaptation, complexity matching, control, complex periodicity, biofeedback

## Abstract

A recent experiment proves the therapeutic effect of arm-in-arm walking, showing that if an aged participant walks in close synchrony with a young companion, the complexity matching effect results in the restoration of complexity in the former. A clear manifestation of complexity restoration is a perfect synchronization. The authors of this interesting experiment leave open two important problems. The first is the measure of complexity that is interpreted as a degree of multifractality. The second problem is the lack of a theoretical derivation of synchronization, which is experimentally observed with no theoretical derivation. The main goal of this paper is to establish a physiological foundation of these important results based on the recent advances on the dynamics of the brain, interpreted as a system at criticality. Criticality is a phenomenon requiring the cooperative interaction of units, the neurons of the brain, and is hypothesized as the main source of cognition. Using the criticality-induced intelligence, we define complexity as a property of crucial events, a form of temporal complexity, and we prove that the perfect synchronization is due to the interaction between the two systems, with the more complex system restoring the temporal complexity of the less complex system. The phenomenon of temporal complexity is characterized by ergodicity breaking that has made it difficult in the past to derive the perfect synchronization generated by complexity matching. For this reason, we supplement the main result of this paper with a comparison between complexity matching and complexity management.

## 1. Introduction: Walking Together as a Form of Therapeutic Synchronization

**Figure 1:**
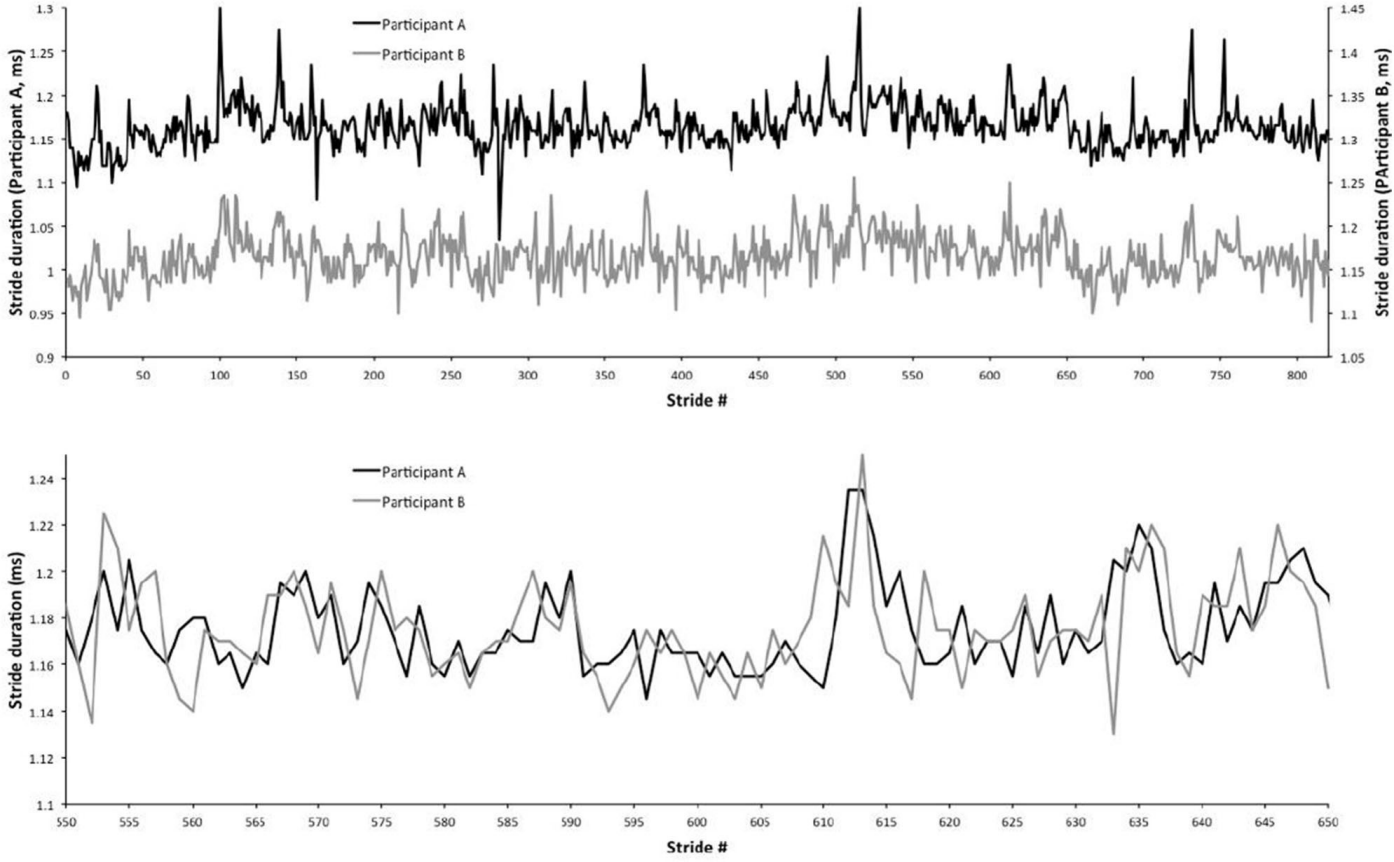
Experimental walking synchronization. The top panel shows two distinct gait trajectories for two human subjects walking together. The bottom panel shows the same trajectories, but overlapping them to emphasize their synchronization. This figure is derived from Almurad et al. (2017), with permission.

Walking in synchrony is a subject of significant interest for its therapeutic effects (Zivotofsky and Hausdorff, 2007; Engelhard, 2018). A remarkably interesting result is illustrated in Almurad et al. (2018), a sequel to the earlier work of Almurad et al. (2017). Senior individuals, with problems in walking and balance, interpreted as a lack of physiological complexity, participated in a longitudinal training program of synchronized walking, with young experimenters. The authors observed a restoration of complexity in the senior participants after 3 weeks, and this effect persisted for at least 2 weeks beyond the end of the training program. Recovering complexity in walking was signaled by synchronization between a senior patient and a youthful experimenter. Figure 1 illustrates the synchronization effect that we intend to recover with simple computational rules by implementing the complexity matching principle (CMP).

The computational prescriptions that we adopt to recover the experimental results of Almurad et al. (2017) and Almurad et al. (2018) are based on neurophysiological arguments that are expected to shed light into the connection between the experimentally observed synchronization and brain dynamics. The synchronization model is reminiscent of the theory of phase-locked modes of Kelso et al. (1987), which in turn, rests on the theoretical perspective of *synergetics* (Haken, 1983) explained in the book by Kelso (1995). However, the coupling between the two interacting systems implies the cooperative action of many units, the neurons of the brain. The present paper designs the phase rearranging selected by one system to establish synchronization with the other, as a consequence of a self-organization process, again reminiscent of synergetics. However, here we draw special attention to fitting the condition of temporal complexity and ergodicity breaking. These two important conditions reflect the recent experimental and theoretical work in the field of neurophysiology, quoted herein to clarify the steps required to reproduce the experimentally observed synchronization of Almurad et al. (2017) and Almurad et al. (2018).

A relevant example of the connection between the model adopted herein and the neurophysiology literature is the use of subordination theory. Bohara et al. (2018) modeled the dynamics of the brain in such a way as to establish a bridge between the nearly coherent oscillations hypothesized by the observation of brain waves and the rapid transition processes, which are compelling results of the recent analysis of *EEGs*. Subordination theory rests on the assumption that coherence is a property of operational time, intimately related to the function of the body. The transition from operational time to clock time is accomplished by introducing crucial events, either visible or invisible, so as to generate a frequency spectrum for time series variability. The spectrum is characterized by 1/*f* noise in remarkably good agreement with experimental observation.

The crucial events are characterized by a complexity index μ, ranging from the value μ = 2, corresponding to the greatest complexity, to the value μ = 3, at the border with the region of ordinary statistical physics, thereby representing the condition of least complexity and greatest pathology. We propose a prescription to couple two different complex-periodic systems, one representing a healthy person and the other representing a sick patient. We prove that as a result of this coupling, the healthy complex system transfers its temporal complexity and its periodicity to the pathological system and the subsequent transfer of information. As a result of this therapeutic process, synchronization between the two systems emerges.

Note that the synchronization depicted in Figure 1 is the result of an experiment while the results shown in Figure 6, which are remarkably similar to those shown in Figure 1, is the result of the coupling between a system with μ close to 3, representing the senior patient, and a system with μ close to 2, representing the young experimenter. Almurad et al. (2017) and Almurad et al. (2018) make the plausible conjecture that their experimental result is a manifestation of the phenomenon of complexity matching, namely the phenomenon of maximal transfer of information between two systems with the same complexity. This special condition of information transfer is made clear by the almost ideal synchronization, which is obtained by increasing the complexity of the senior subject while the complexity of the healthy youth remains unchanged. These authors however leave open the definition of complexity that, according to them, can be measured by multi-fractality.

In line with the theory of Mahmoodi et al. (1990) showing that multifractality is generated by crucial events at criticality, the main goal of the present manuscript is to prove that the complexity index μ is the proper measure of complexity. To make the significance of achieving this goal more apparent, we supplement this result with a proper revision of the connection between complexity matching and complexity management.

## 2. Method

To address the ambitious purpose of explaining synchronization, we adopt subordination theory (Sokolov, 2002). This theory allows us to combine rhythm, which is a fundamental property of biological processes (Winfree, 2001) with non-rhythmic crucial events. The crucial events are organization rearrangements, or renewal failures observed in the brain (Paradisi et al., 2016) and are closely related to the phenomena of intermittency (Metzler et al., 2014).

The time distance τ between consecutive crucial events is described by a waiting-time probability density function (PDF) ψ(τ) with an inverse power law (IPL) structure:

(1) $$\begin{array}{l} \psi \left(\tau \right)\propto \frac{1}{\tau^{\mu }} \end{array}$$

with the IPL index in the interval:

(2) $$\begin{array}{l} 1<\mu <3. \end{array}$$

These crucial events are the source of aging and of non-stationary correlation functions (Metzler et al., 2014), and aging is perennial if μ < 2. Earlier research work shows that the human brain operates in the region 2 < μ < 3 (Bohara et al., 2017), where the first moment < τ > of the PDF ψ(τ), is finite. Therefore, we focus on this condition to establish a connection between renewal events and rhythm. Rhythm is a property of the operational time *n* (Sokolov, 2002) and corresponds to harmonic motion:

(3) $$\begin{array}{l} x\left(n\right)=cos\left(\Omega n\right). \end{array}$$

Herein we refer to this harmonic motion by means of the time period:

(4) $$\begin{array}{l} T=\frac{2\pi }{\Omega }, \end{array}$$

as well as the frequency Ω.

In clock time, according to subordination theory, *x*(*t*) is given by (Bohara et al., 2017) :

(5) $$\begin{array}{l} x\left(t\right)=\overset{\infty }{\underset{n=0}{\sum }}\int_{0}^{t}dt^{\prime }\psi_{n}\left(t^{\prime }\right)\text{Ψ}\left(t-t^{\prime }\right)cos\Omega n, \end{array}$$

where $\psi_{n}\left(t^{\prime }\right)$ is the PDF corresponding to the occurrence of the *n*-th crucial events at time *t*′. From time *t*′ to time *t* no further crucial event occurs. This constraint is established by Ψ(*t* − *t*′), with Ψ(*t*) being the survival probability associated to the waiting–time PDF ψ(*t*). Note that *x*(*t*) of Equation (5) can be interpreted as being the harmonic motion of Equation (3) made complex through the transform *n* → *t*.

A clear sign of the complexity of *x*(*t*) is that its power spectrum is characterized (Bohara et al., 2017) by the IPL formula:

(6) $$\begin{array}{l} S\left(f\right)\propto \frac{1}{f^{\beta }}, \end{array}$$

where the spectral IPL index is:

(7) $$\begin{array}{l} \beta \equiv 3-\mu . \end{array}$$

Note that when μ = 2, *S*(*f*) of Equation (6) yields β = 1, namely, the ideal 1/*f*-noise as found by Allegrini et al. (2009) to be produced by a healthy human brain.

**Figure 2:**
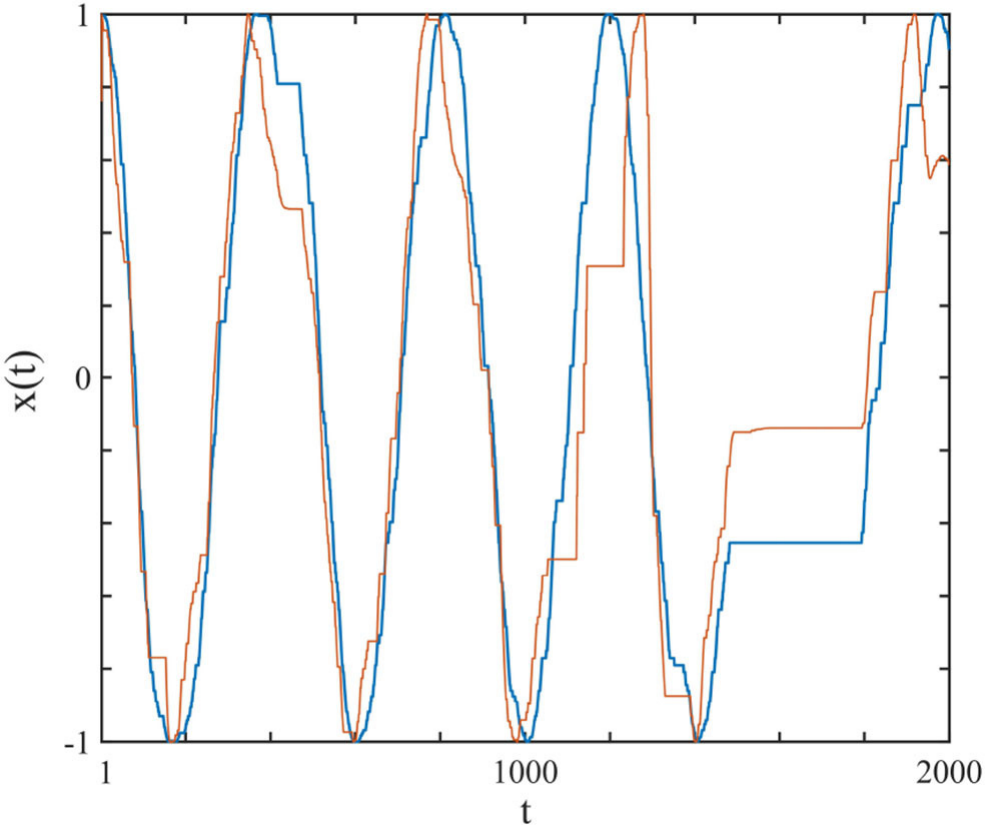
The curves show the *x*(*t*) of the driving system (blue), modeled by a subordinated cosine wave with μ_1_ = 2.7 and Ω_1_ = 2π/200, which was connected (uni-directional) with perturbation strength *r*_2_ = 0.1 to the driven system (red), with μ_2_ = 2.3 and Ω_2_ = 2π/100. The connection is realized using Equation (9). Ensemble size = 1.

In Figure 2 we show one complex system driving another and synchronization is achieved. Herein we explain how this synchronization is realized through the study of two complex systems, *S*_1_ and *S*_2_, with their respective frequencies and complexity parameters, Ω_1_, μ_1_ and Ω_2_, μ_2_. Note that here we use the IPL indices as measures of the systems' complexity.

At time *t* the subordination process yields for *S*_1_:

(8) $$\begin{array}{l} x_{1}\left(t\right)=cos\left(\Omega_{1}n_{1}\left(t\right)\right). \end{array}$$

and for *S*_2_:

(9) $$\begin{array}{l} x_{2}\left(t\right)=cos\left(\Omega_{2}n_{2}\left(t\right)\right). \end{array}$$

Of course, in the absence of coupling *n*_1_(*t*) ≠ *n*_2_(*t*). Let us assume that *S*_1_ is the driving and *S*_2_ the driven system. The coupling is realized through the intelligent response of *S*_2_, which tries to compensate for the difference between *x*_1_(*t*) and *x*_2_(*t*) by rearranging the phase according to the prescription;

(10) $$\begin{array}{l} \Phi \left(t+1\right)=\Phi \left(t\right)-r_{2}K\left(t\right)sign\left\{sin\left(\Omega_{2}n_{2}\left(t\right)+\Phi \left(t\right)\right)\right\}, \end{array}$$

where the difference between the driven and driver systems is:

(11) $$\begin{array}{l} K\left(t\right)\equiv 2\left[x_{1}\left(t\right)-x_{2}\left(t\right)\right]. \end{array}$$

This means that the driven system is aware of whether the difference Δ_*a*_(*t*) ≡ *x*_1_(*t*) − *x*_2_(*t*) is positive or negative. In addition it also knows the gradient Δ_*b*_(*t*) ≡ ∂*x*_2_/∂*n*_2_∝ − *sin*(Ω_2_*n*_2_(*t*), namely the derivative of *x*_2_ with respect to operational time. The complex driven system *S*_2_ increases or decreases its phase Φ(*t*) depending on the sign and magnitude of the product Δ_*a*_(*t*)Δ_*b*_(*t*).

Equation (10) is a generalization of the swarm intelligence prescription adopted in earlier work (Turalska et al., 2009; Vanni et al., 2011) and is the learning process in our algorithm which enables the driven system to continuously adopt to the driving system, thereby creating complexity matching between them. In Figure 2 we illustrate the typical synchronization obtained by assigning to the driving system μ_1_ = 2.7, Ω_1_ = 2π/200 and to the driven system μ_2_ = 2.3, Ω_2_ = 2π/100.

**Figure 3:**
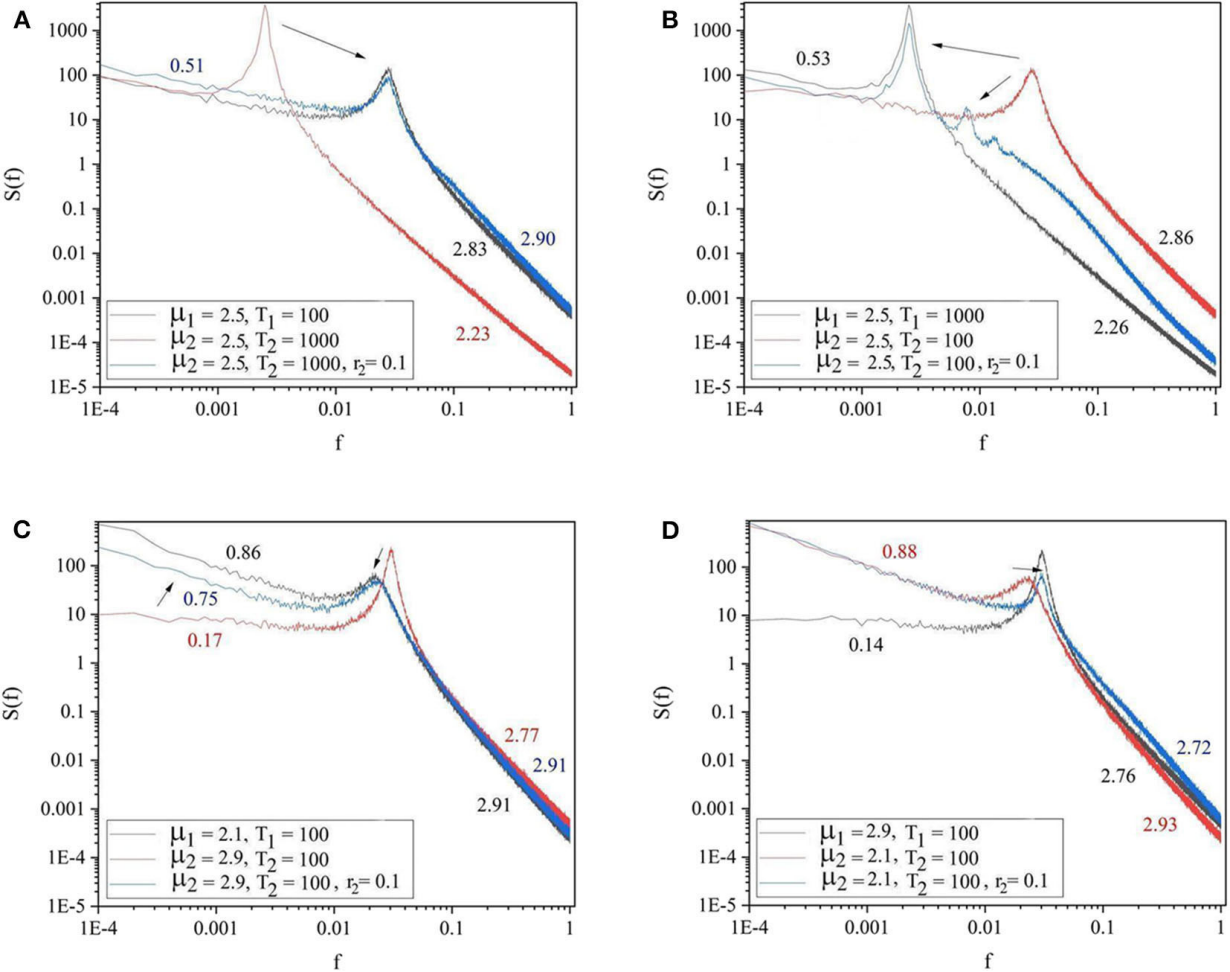
The spectra of the driving system (black curve), driven system before connection (red curve), and driven system after connection (blue curve). **(A)** μ_1_ = 2.5, Ω_1_ = 2π/100, μ_2_ = 2.5, Ω_2_ = 2π/1, 000, *r*_2_ = 0.1. **(B)** μ_1_ = 2.5, Ω_1_ = 2π/1, 000, μ_2_ = 2.5, Ω_2_ = 2π/100, *r*_2_ = 0.1. **(C)** μ_1_ = 2.1, Ω_1_ = 2π/100, μ_2_ = 2.9, Ω_2_ = 2π/100, *r*_2_ = 0.1. **(D)** μ_1_ = 2.9, Ω_1_ = 2π/100, μ_2_ = 2.1, Ω_2_ = 2π/100, *r*_2_ = 0.1. *L* = 10^5^. Ensemble size = 100.

Each panel of Figure 3 shows the spectra of the driving system (black curve) and of the driven system before (red curve) and after (blue curve) connection. Panels 3A,B refer to the cases where both driving and driven systems have the same complexity μ_1_ = μ_2_ = 2.5, but different periodicities. In panel 3A the driving system has the lower periodicity (Ω_1_ = 2π/100 and Ω_2_ = 2π/1, 000) while in panel 3B the driven system has the higher periodicity (Ω_1_ = 2π/1, 000 and Ω_2_ = 2π/100). The results depicted in these two panels reveal that the driven system adopts the periodicity of the driving systems. The driven system with higher periodicity shifts its periodicity to that of the driving system (3A). Panel 3B shows that the driven system with lower periodicity adopts the periodicity of the driver, as well as, the other embedded oscillation modes. The remaining two panels of Figure 3 are cases where both driving and driven systems have the same periodicity (Ω_1_ = Ω_2_ = 2π/100), but different complexity indices. In Panel 3C the driver has higher complexity (lower complexity index) (μ_1_ = 2.1 < μ_2_ = 2.9) while in Panel 3D the driver has lower complexity (higher complexity index) (μ_1_ = 2.9 > μ_2_ = 2.1). These panels show that the less complex system could adapt to both the complexity index and periodicity of the more complex driven system (3C). By contrast, Panel 3D shows that the more complex driven system does not adapt to the complexity index and periodicity of the less complex driver.

**Figure 4:**
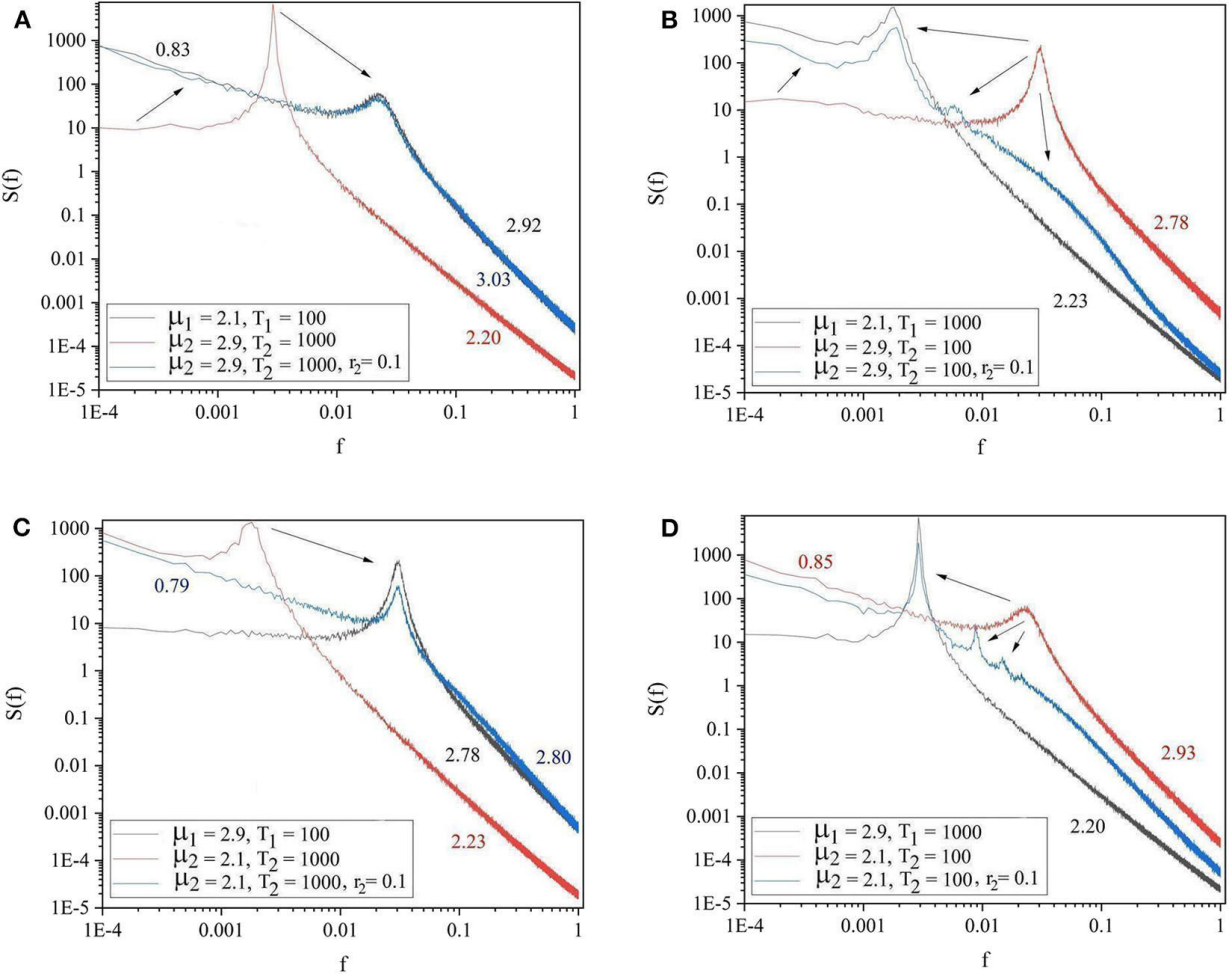
The spectra of the driving system (black curve), driven system before connection (red curve) and driven system after connection (blue curve). **(A)** μ_1_ = 2.1, Ω_1_ = 2π/100, μ_2_ = 2.9, Ω_2_ = 2π/1, 000, *r*_2_ = 0.1. **(B)** μ_1_ = 2.1, Ω_1_ = 2π/1, 000, μ_2_ = 2.9, Ω_2_ = 2π/100, *r*_2_ = 0.1. **(C)** μ_1_ = 2.9, Ω_1_ = 2π/100, μ_2_ = 2.1, Ω_2_ = 2π/1, 000, *r*_2_ = 0.1. **(D)** μ_1_ = 2.9, Ω_1_ = 2π/1, 000, μ_2_ = 2.1, Ω_2_ = 2π/100, *r*_2_ = 0.1. *L* = 10^5^. Ensemble size = 100.

The panels of Figure 4 show the four general conditions where the driving and driven systems have different parameters for both complexity and periodicity. The results of these figures follow the same patterns as those of Figure 3. Notice there are also signs of the extra oscillation modes between the driving and the driven frequency illustrated in Figures 3B, 4B,D.

**Figure 5:**
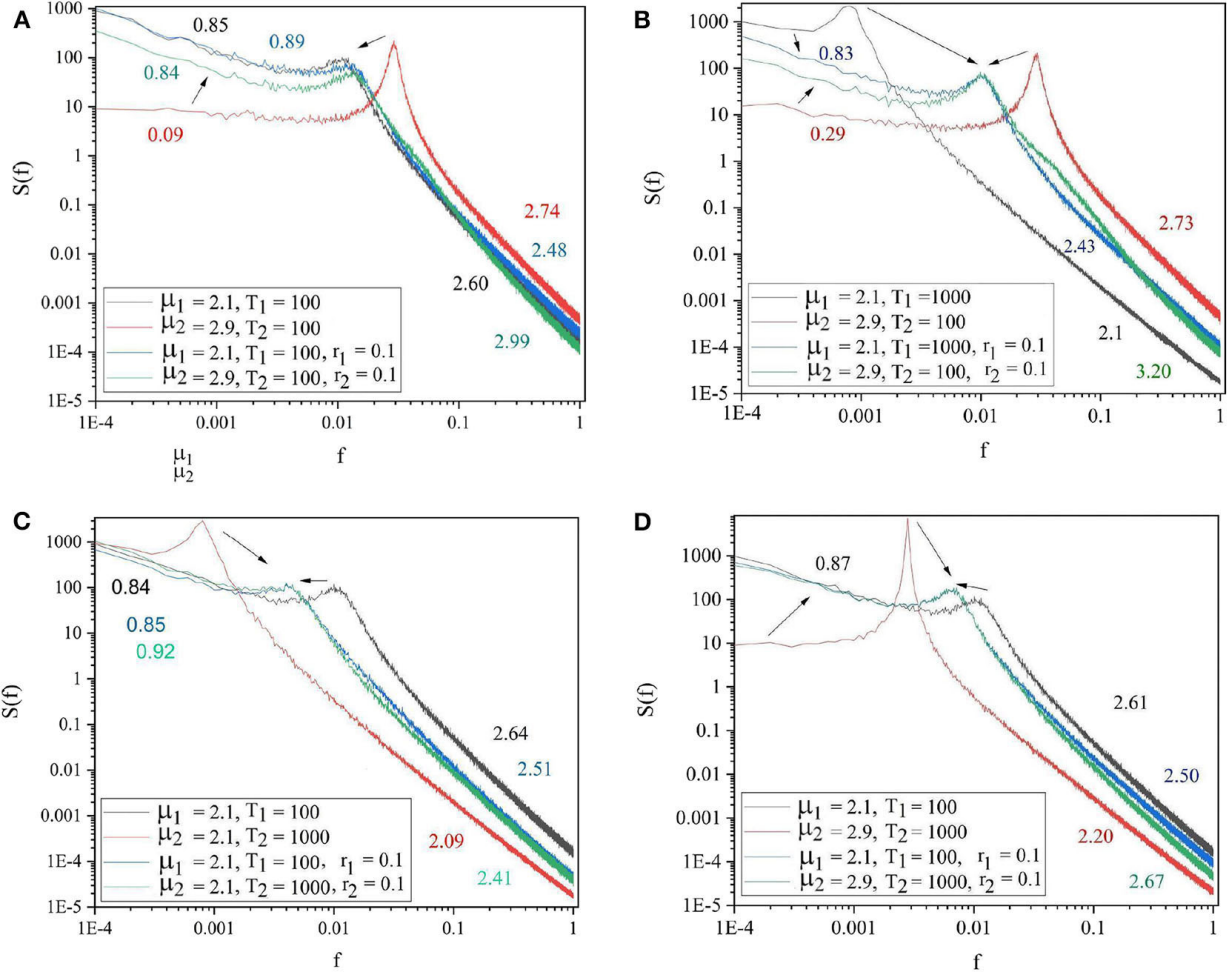
The spectra of the two systems at isolation (*S*_1_: black and *S*_2_: red curves) and after being connected back to back (blue and green curves, respectively). **(A)** μ_1_ = 2.1, Ω_1_ = 2π/100, μ_2_ = 2.9, Ω_2_ = 2π/100. **(B)** μ_1_ = 2.1, Ω_1_ = 2π/1, 000, μ_2_ = 2.9, Ω_2_ = 2π/100. **(C)** μ_1_ = 2.1, Ω_1_ = 2π/100, μ_2_ = 2.1, Ω_2_ = 2π/1, 000. **(D)** μ_1_ = 2.1, Ω_1_ = 2π/100, μ_2_ = 2.9, Ω_2_ = 2π/1, 000. *r*_1_ = *r*_2_ = 0.1. *L* = 10^5^. Ensemble size = 100.

Of great importance for the therapeutic effect of walking together is the condition where *S*_1_ is influenced by *S*_2_ in the same way. We refer to this condition as back–to–back, also known as bi-directional information exchange. To realize the back–to–back condition, as we shall subsequently see that we need to introduce the new parameter *r*_1_, which defines the intensity of the influence of S_2_ on S_1_. The panels in Figure 5 show the cases where two systems are connected back–to–back. The systems with lower complexity (μ_2_ = 2.9) improved their complexity from μ_1_ = 2.9 to μ_1_ = 2.1 and both systems adopted a frequency between the initial effective frequencies.

Here we have to stress that the perturbing system is quite different from the external fluctuation that was originally adopted to mimic the effort generated by a difficult task (Corell, 2008; Grigolini et al., 2009). In that case, according to Heidegger's phenomenology (Dotov et al., 2010) the transition from *ready-to-hand* to *unready-to-hand* makes the IPL index μ depart from the 1/*f*-noise condition μ = 2 (Corell, 2008; Grigolini et al., 2009) so as to reach the Gaussian border μ = 3 and to go beyond it. Here the perturbation is characterized by an intense periodicity, and while it does not change the complexity of the perturbed network very much, it does transfer its own periodicity.

The theory developed herein may shed light on the crucial role of cooperation. Recent psychological research on collective intelligence (Woolley et al., 2010) shows that cooperative interactions between the members of a group may improve the global intelligence of that group. To realize a condition that is close to that of the paper of Woolley et al. (2010), we study the case where S_1_ is influenced by S_2_ in the same way S_2_ is influenced by S_1_. As a result of this mutual interaction, we have $\mu_{1}\to \mu_{1}^{\prime }$and $\mu_{2}\to \mu_{2}^{\prime }$. When μ_1_ < μ_2_ we expect the shifted complexity indices to lie in the interval:

(12) $$\begin{array}{l} \mu_{1}<\mu_{1}\prime <\mu_{2}\prime <\mu_{2}. \end{array}$$

Figure 5 shows that $\mu_{1}^{\prime }\approx \mu_{1}$, thereby suggesting that the system with higher complexity does not perceive its interaction with the other system as a difficult task, which would force it to increase its own μ (Corell, 2008; Grigolini et al., 2009), while the less complex system has a sense of relief. We interpret this result as an important property that should be the subject of psychological experiments to shed light on the mechanisms facilitating the teaching and learning process.

The term “intelligent” that we use herein is equivalent to assessing a system to be as close as possible to the ideal condition μ = 2, corresponding to the ideal 1/*f* noise. In this sense, two very intelligent systems are the brain and heart that, when healthy, share the property of a μ being close to 2. The present paper, therefore, provides a rationale for (an explanation of) the synchronization between heart and brain time series (Pfurtscheller et al., 2017) and shows that the concept of resonance, based on tuning the frequency of the stimulus to that of the system being perturbed, may not be appropriate for complex biological systems. Resonance is more appropriate for a physical system, where the tuning has been adopted over the years for the transport of energy, not information. The widely used therapies resting on bio-feedback (Lin and Li, 2018), are the subject of appraisal (Papo, 2019), and the present results may contribute to making therapeutic progress by establishing their proper use.

## 3. Supporting Information

### 3.1. Walking Together

**Figure 6:**
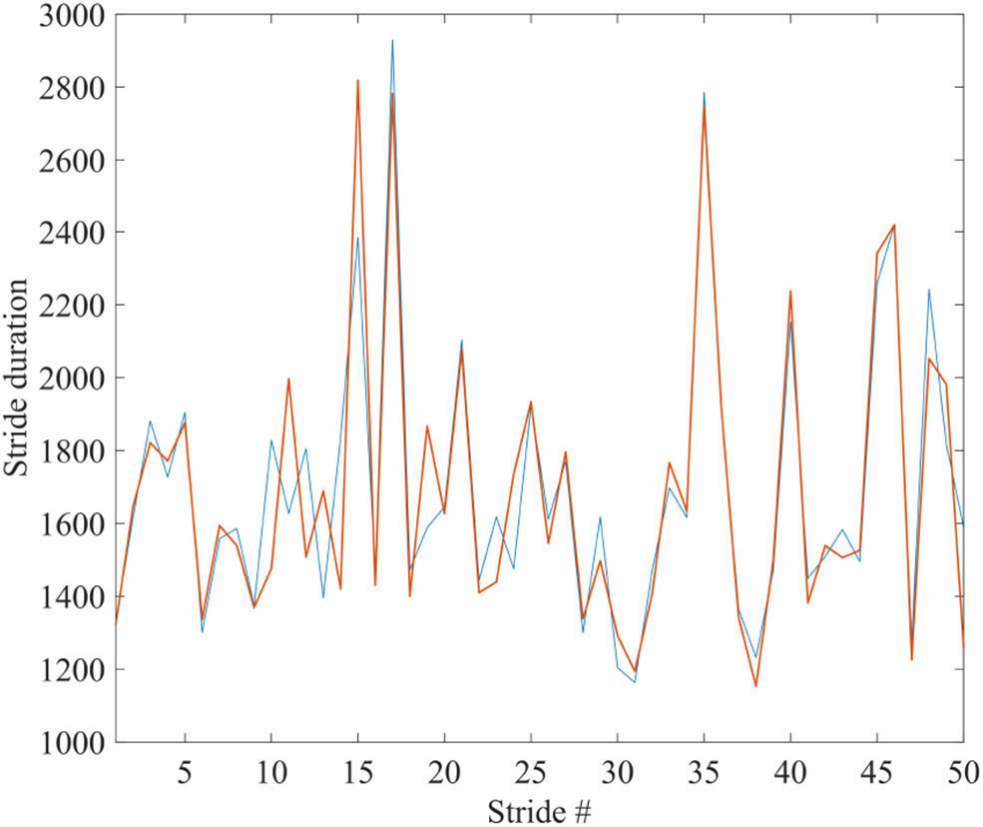
The blue and red curves show the duration times between the strides of x(t) of two systems, being connected back to back; corresponding to the spectra of Figure 4B: μ_1_ = 2.1, Ω_1_ = 2π/1, 000, μ_2_ = 2.9, Ω_2_ = 2π/100, *r*_1_ = *r*_2_ = 0.1. Ensemble size = 1.

To facilitate the appreciation of the similarity between the complexity matching prescription observed herein and the walking synchronization of the paper of Almurad et al. (2017), we invite the readers to examine the experimental results of Figure 1. We used numerical results, of the same kind as those illustrated in Figure 2 of the text, properly modified to connect the two trajectories back–to–back. To make the qualitative similarity with the results of the experiment of the paper of Almurad et al. (2017) more evident, we adopt the same prescription as that used by Almurad et al. We interpret the time interval between consecutive crossings of the origin, *x* = 0, as the time duration of a stride of *x*(*t*). Figure 6 illustrates the result of this procedure. This remarkably good qualitative agreement between Figures 1, 6 supports the efficiency of the complexity matching approach used herein. This procedure can also be used to explain the synchronization between the heart and brain found empirically by Pfurtscheller et al. (2017).

### 3.2. Beyond Complexity Management

We also show how the method of the present paper works when applied to experimental data to evaluate the cross-correlation between the driven and the driving complex networks, going beyond the limitations of the research work on complexity management (Aquino et al., 2011; Piccinini et al., 2016). Complexity management is very difficult to observe. It is based on ensemble averages, thereby requiring the average over many identical realizations (Aquino et al., 2011). In the case of experimental signals of physiological interest, for instance, on the brain dynamics, taking an ensemble average is not possible. The theory presented herein makes it possible to evaluate the correlation between the driving and the driven system using a single realization of the time series. We stress that while complexity management (Aquino et al., 2011) does not affect the IPL index μ of the interacting complex networks, the theory of this paper, as shown by Figure 4, affords important information on how the cooperative interaction makes the unperturbed values of μ change as a consequence of the interaction.

**Figure 7:**
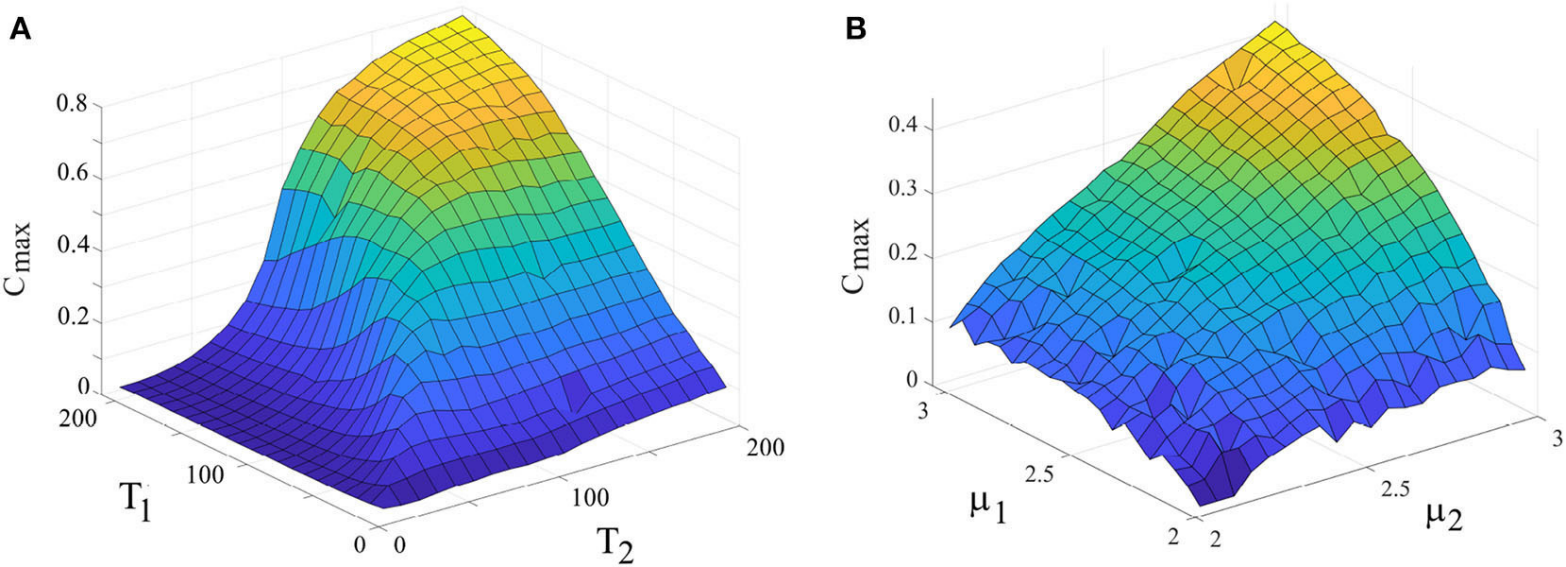
**(A)** Dependence of *C*_*max*_ (as a measure for synchronization) on the periodicity of the drive and driven systems. μ_1_ = μ_2_ = 2.5. *r*_2_ = 0.025, *L* = 5 × 10^7^. **(B)** Dependence of *C*_*max*_ on the complexity index of the drive and driven systems. *T*_1_ = *T*_2_ = 50, *r*_2_ = 0.025, *L* = 5 × 10^7^. Ensemble size = 1.

Panel 7A illustrates the maximum value of the cross-correlation function *C*_*max*_ vs. periodicity of the driver and the driven systems connected, uni-directionally, *r*_2_ = 0.025, while keeping their complexity index equal: μ_1_ = μ_2_ = 2.5. High values of *C*_*max*_ corresponds to the strong adaptability of the driven system to the driving system. This figure shows that when the driven system has the periodicity similar to that of the driver, its adaptation is maximum. Panel 7B shows the cross-correlation function, *C*_*max*_ vs. complexity index of the driver and driven systems connected, uni-directionally, *r*_2_ = 0.025, while keeping their periodicity equal: Ω_1_ = Ω_2_ = 2π/50. Notice that there is no ensemble averaging done in producing Figure 7. A driven system with lower complexity (higher μ) adapted more to the driving system than does a driven system with higher complexity.

**Figure 8:**
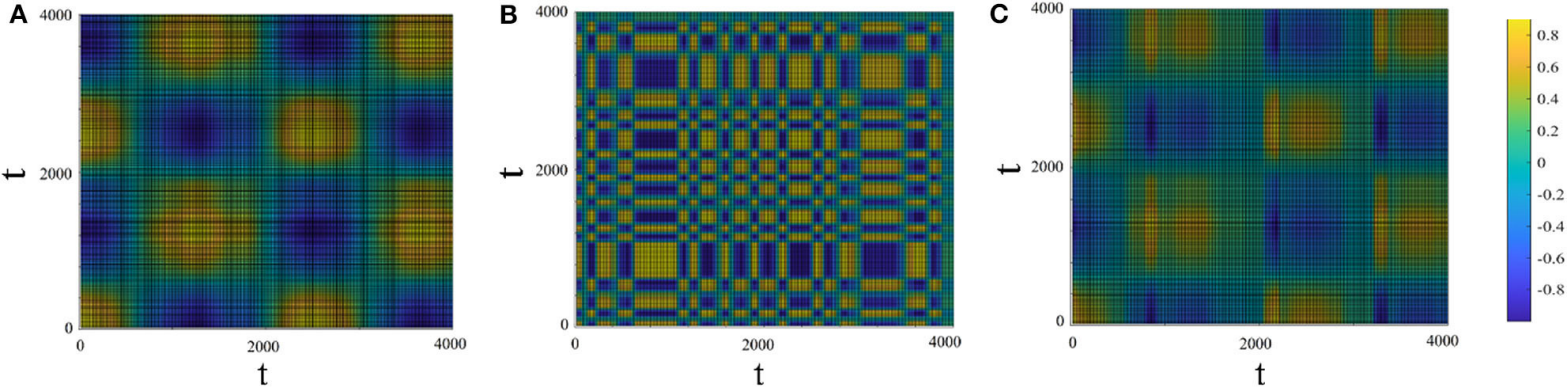
Recurrent plots. The x and y axes are time. The colors in the panels correspond to the values of *x*_1_(*t*_1_) × *x*_1_(*t*_2_) (**A**; driving system), *x*_2_(*t*_1_) × *x*_2_(*t*_2_) (**B**; driven system before connection) and *x*_1_(*t*_1_) × *x*_2_(*t*_2_) (**C**; cross-recurrence between the driving and the driven system after connection). Drive system: μ_1_ = 2.9, *T*_1_ = 1, 000. Driven system: μ_2_ = 2.1, *T*_2_ = 100, *r*_2_ = 0.1.

Figure 8 shows the recurrent plots which provide a way to visualize the changes in the periodic nature of the driven system before and after being connected to the driving system. In Panels 8A,B the colors indicate the value of *x*_1_(*t*_1_) × *x*_1_(*t*_2_) and *x*_2_(*t*_1_) × *x*_2_(*t*_2_) for the driver (with μ_1_ = 2.9, *T*_1_ = 1, 000) and driven (with μ_2_ = 2.1, *T*_2_ = 100) systems, respectively. Panel 8C shows the cross–recurrence between the driving and driven systems after connection (*r*_2_ = 0.1). Panel 8C shows that the driven system adapted the complex periodicity of the driving system and in addition gained some extra oscillation modes in between (corresponding to panel 8B).

## 4. Complexity, Information, and Conclusions

In the recent literature on self-organization (see e.g., Gershenson and Fernández, 2012), the emergence of complexity is interpreted as corresponding to information reduction. Variety increases with a complex system performing multitask actions and decreases with a complex system focusing on a single task (Bar-Yam, 2004). More recent work confirms this property in sociological systems (Zhang et al., 2018), while it is well-known that it holds true for physiological processes (Peng et al., 1995; Struzik et al., 2004). The hypothesis of self-organization has been known and used in biology for nearly half a century (Eigen, 1971; Thompson and McBride, 1974) [see also Chapter 5 of Eigen's important book Eigen (2013)].

### 4.1. Information Reduction

The entropic approach used to deal with crucial events is the Kolmogorov-Sinai (KS) entropy *h*_*KS*_ (Ignaccolo et al., 2001), which is well-described by the formula

(13) $$\begin{array}{l} h_{KS}=z\left(2-z\right)ln2, \end{array}$$

where $z\equiv \frac{\mu }{\mu -1}$. Equation (13) indicates that the KS entropy vanishes at *z* = 2 and it remains equal to 0 in the entire infinite interval 2 < *z* < ∞ (μ < 2). Allegrini et al. (2003) noticed that *z* = 1, corresponding to μ = ∞, is the condition of total randomness, namely, the case where an infinitely large amount of information is necessary to control the system. The condition *z* = 1.5, corresponding to μ = 3, makes the sequence of crucial events compressible, namely, it reduces the amount of information necessary to control the system, and finally, the *KS* entropy vanishes when μ < 2. This is a region characterized by the diverging value of the mean waiting time < τ >.

The recent generalization to the mechanism of self–organized criticality given by self–organized temporal criticality (*SOTC*) (Mahmoodi et al., 2017) generates crucial events with μ < 2, and, albeit a form of self–organization yielding values of μ in the interval 2 < μ < 3 is not yet known, we make the plausible conjecture that complex processes that are experimentally proven to generate crucial events in this interval as well as in the interval 1 < μ < 2, are the result of a process of self-organization. The condition *z* > 2 (μ < 2) is where Korabel and Barkai (2009) had to modify Equation (13) leaving this expression unchanged for 1 < *z* < 2 and making it increase from the vanishing value with *z* > 2. Actually, *KS* entropy is a Lyapunov coefficient, and Korabel and Barkai defined the Lyapunov coefficient for *z* > 2, by comparing the rate of departure between two trajectories moving from very close initial conditions to *t*^μ−1^, rather than to *t*, as correctly done for *z* < 2. This means that the region *z* > 2 (μ < 2) is not fully deterministic, but the amount of information necessary to control the system is drastically reduced.

### 4.2. Requisite Variety

Ivanov et al. (1999) noticed that healthy heartbeats have variability that makes it impossible to adopt the conventional method of analysis of anomalous scaling based on the stationary assumption. Consequently, they made the assumption of a scaling fluctuation that led them to adopt a multifractal approach. Their proposal turned out to be extremely successful and was adopted to distinguish healthy heartbeats from heart failure heartbeats (Ivanov et al., 1999). Allegrini et al. (2002) examined the data from the same cohort of patients studied in Ivanov et al. (1999) using the crucial events defined herein and found that healthy patients have a μ very close to 2, which makes the *KS* entropy vanish. They also conjectured that self-organization generating crucial events may also be the generator of multifractality. This last conjecture has been fully confirmed in the recent work of Bohara et al. (2017) and Mahmoodi et al. (1990).

Of remarkable importance for the requisite variety issue is the work by Struzik et al. (2004), emphasizing the transition from 1/*f* noise to 1/*f*^2^ noise as a manifestation of variability suppression. Healthy heart physiology is based on the balance between the conflicting action of the sympathetic and parasympathetic nervous systems, thereby resulting in the ideal 1/*f* noise for healthy individuals and in the 1/*f*^2^ noise for pathological individuals. This condition is examined herein with the help of Figure 1. The *SOTC* time series of the paper of Mahmoodi et al. (2017) yields μ < 2. We examined the case of μ moving into the interval 2 < μ < 3 using subordination to regular oscillatory motion, a phenomenological way of combining crucial events and periodicity. We believe that *SOTC* can be extended to this condition, and have confidence that future work will realize this important goal. We see that for *f* → 0, the IPL spectra $S_{p}\left(f\right)\propto 1/f^{\beta }$, has β = 3 − μ. The ideal condition of 1/*f* noise is realized when μ = 2. The transition from 1/*f* noise to white noise is realized by increasing μ from the ideal value μ = 2 to the value μ = 3 and beyond. In the presence of periodicity, though, the 1/*f* noise region can also be affected by moving the periodicity peak from right to left, in such a way as to make the 1/*f*^2^ noise the dominant contribution to the spectra in accordance with the experimental observation of Struzik et al. (2004).

### 4.3. Lack of Difficult Task Perception

The results of Figure 5 showing μ_1_′ ≈ μ_1_, as earlier stated, suggest that the system with higher complexity does not perceive the interaction with the less complex system as a difficult task. This is an indication that the dynamical model adopted in this paper works at a merely physiological level with no direct influence on behavior. Further research work is necessary to go beyond the limits of the model of this paper. An interesting example of a valuable direction to follow to realize this extension is afforded by the recent work of Tognoli et al. (2018). The authors of this illuminating paper adopt a multiscale neurocomputational model of social coordination that enables exploration of social self–organization at all levels, from neuronal patterns to people interacting with each other. The theoretical background is afforded by the *synergetics* of Haken (1983) that is based on the contraction over fast irrelevant variables thereby addressing criticality with no attention to the ergodicity breaking role of crucial events. The present paper shows that crucial events should be taken into account.

We believe that in principle this extension can be realized by adopting the payoff arguments of Mahmoodi et al. (2017) in SOTC. In the model of the present paper, the less complex systems have more events and consequently more chances to adapt their phases to the more complex system. Therefore, the less complex network matches the trajectory of the more complex network with no need to go beyond the physiologic level. If the two systems were connected in a way that the payoff of each system depended also on the performance of the other, then the more complex could increase its μ to help the less complex to decrease its μ, but together they could reach maximum performance. This may have the effect of explaining the earlier mentioned results on the collective intelligence of Woolley et al. (2010).

Adopting the distinction between neurophysiologic and sociologic level (Tognoli et al., 2018), interpreted as two distinct complex systems, we should be able to take into account the perception of task difficulty, going beyond the limitations of the model adopted herein. A remarkable example of a problem that would be settled using this extension of the model of the present paper is given by Tuladhar et al. (2018). Tuladhar et al. analyzed the heartbeats of subjects practicing meditation and found that this has the effect of generating additional coherence and increasing the executive control, while moving μ from low to high values, a property adopted in the earlier work of Allegrini et al. (2009) to explain the Corell effect (Corell, 2008), interpreted as a consequence of the perception of task difficulty. Adopting a proper extension of the present model, the improved executive control would be interpreted as the behavioral and neurologic systems reaching the level of maximum performance.

Another important problem requiring further theoretical advances is the persistence of complexity restoration. From a theoretical point of view, this is an open problem. In fact, the actions of the system with less complexity are determined by subordination to harmonic processes with the transition from operational to clock time being determined by a waiting–time PDF with a fixed value of the complexity parameter μ. On the basis of the statistical analysis of real *EEG*′*s* and *EKG*′*s* this parameter has been assigned a value close to 2 to simulate healthy systems and close to 3 to simulate systems affected by pathologies. These values of μ are the result of a dynamic interaction between the units of the complex systems. The papers of Turalska et al. (2009) and Vanni et al. (2011) show that the intelligent behavior of a system is determined by a control parameter *K*, the strength of the interaction between the system's units. To assess the persistence of complexity restoration we would need a theory where *K* is not fixed but may change according to the interaction with the environment. The *SOTC* of Mahmoodi et al. (2017) is based on the assumption that the search for an optimal payoff has the effect of changing the control parameter *K*, with the pathological behavior being determined by a top-down rather than bottom-up approach to complexity. Therefore, a theory to assess the persistence of the recovery would require an extended form of *SOTC*, which balances the top-down with the bottom-up effects. On the other hand, the experimental results of Almurad et al. (2018) on the rehabilitation protocol indicate that there may be a form of persistence making the new training process easier and faster. But this is not firmly proved, requiring further experiments. Therefore, we are inclined to conclude that the assessment of this important issue will be the result of further research work, at both experimental and theoretical level.

Finally, we conclude by stressing that the surprisingly accurate synchronization of the walking together process ought not to be confused with either chaos synchronization or resonance. In fact, chaos synchronization requires finite Lyapunov coefficients and resonance requires frequency tuning. Complex systems with μ very close to the ideal condition μ = 2, where the traditional Lyapunov coefficient vanishes, have the effect of transferring their temporal complexity to systems with higher values of μ. The numerical results show that, although communication through frequencies still exists (bottom panel of Figure 5), the action of crucial events is more important for the transfer of intelligence. Our theoretical approach is based on the essential role of crucial events. The crucial events with μ becoming closer to μ = 2 are generators of multifractality, as pointed out in the work of Bohara et al. (2017) and Mahmoodi et al. (1990). Thus, our prediction that the walker with μ close to 2 attracts the μ of the walker close to the Gaussian region μ = 3 can be interpreted as transmission of multifractality from the healthy to the pathological walker in a surprising agreement with the recent experimental result of Almurad et al. (2018).

## Data Availability Statement

The raw data supporting the conclusions of this article will be made available by the authors, without undue reservation.

## Author Contributions

KM modeled and performed the computations. PG and BW supervised the findings of this work. All authors provided the critical feedback and helped to shape the research, analysis, and manuscript.

## Conflict of Interest

The authors declare that the research was conducted in the absence of any commercial or financial relationships that could be construed as a potential conflict of interest.
